# Daratumumab combined with dexamethasone and lenalidomide or bortezomib in relapsed/refractory multiple myeloma (RRMM) patients: Report from the multiple myeloma GIMEMA Lazio group

**DOI:** 10.1002/jha2.359

**Published:** 2022-01-15

**Authors:** Francesca Fazio, Luca Franceschini, Valeria Tomarchio, Angela Rago, Maria Grazia Garzia, Luca Cupelli, Velia Bongarzoni, Alessandro Andriani, Svitlana Gumenyuk, Agostino Tafuri, Agostina Siniscalchi, Alfonso Piciocchi, Paolo De Fabritiis, Luca De Rosa, Tommaso Caravita di Toritto, Ombretta Annibali, Maria Cantonetti, Maria Teresa Petrucci

**Affiliations:** ^1^ Department of Translational and Precision Medicine, Hematology Azienda Policlinico Umberto I Sapienza University of RomeSapienza Università di Roma; ^2^ Transplant Network, Hematology‐Stem Cell Transplant Unit Rome Italy; ^3^ Department of Haematology, University Campus Biomedico Rome Italy; ^4^ ASL RM/A UOSD Ematologia Asl Roma 1 Rome Italy; ^5^ Department of Hematology, Hematology San Camillo Forlanini Hospital Rome Italy; ^6^ Department of Hematology, Hematology Ospedale Sant'Eugenio Rome Italy; ^7^ Department of Hematology San Giovanni‐Addolorata Hospital Rome Italy; ^8^ Department of Hematology Ospedale Fabrizio Spaziani Rome Italy; ^9^ Haematology and Stem Cell Transplant Regina Elena National Cancer Institute Rome Italy; ^10^ Azienda Ospedaliera Sant'Andrea Rome Italy; ^11^ S. Eugenio Hospital Institute of Haematology Rome Italy; ^12^ Data Center, Italian Group for Adult Hematologic Diseases, (GIMEMA) Rome Italy; ^13^ Department of Haematology Ospedale Sant'Eugenio Rome Italy; ^14^ Hematology and Bone Marrow Transplantation Unit Azienda Ospedaliera San Camillo‐Forlanini Rome Italy; ^15^ Department of Hematology, ASL Roma 1 Rome Italy; ^16^ Department of Haematology Campus Bio‐Medico University of Rome Rome Italy; ^17^ Chair of Hematology, Universita Tor Vergata Rome Italy

**Keywords:** immunotherapy, multiple myeloma, relapsed refractory

## Abstract

The multiple myeloma (MM) treatment has changed over the last years due to the introduction of novel drugs. Despite improvements in the MM outcome, MM remains an incurable disease. Daratumumab is a human IgGK monoclonal antibody targeting CD38 with tumor activity associated with immunomodulatory mechanism. In combination with standard of care regimens, including bortezomib (Vd) or lenalidomide (Rd), daratumumab prolonged progression‐free survival (PFS) in patients (pts) with relapsed/refractory multiple myeloma (RRMM) and in new diagnosis MM. We report the data of the MM GIMEMA Lazio group in 171 heavily treated pts who received daratumumab, lenalidomide and dexamethasone (DRd) or daratumumab, velcade and dexamethasone (DVd). The overall response rate was 80%, and the overall survival (OS) and PFS were 84% and 77%, respectively. In addition, pts treated with DRd showed a better median PFS compared to pts treated with DVd, at 12 and 24 months, respectively. The most common hematologic treatment‐emergent adverse events (TAEs) were neutropenia, thrombocytopenia, and anemia. The most common nonhematologic TAEs were peripheral sensory neuropathy and infections. Our data confirmed that DRd or DVd therapy is effective and safe in RRMM pts, and our real‐life analysis could support the physicians regarding the choice of optimal therapy in this setting of pts.

## 1 INTRODUCTION

The treatment of multiple myeloma (MM) has changed over the last decade, and the therapeutic armamentarium of effective anti‐plasma cell drugs has expanded including several novel molecules, such as proteasome inhibitors (PIs) and immunomodulatory drugs (IMiDs) [1, 2].The incorporation of these novel agents into the standard of care regimens and use of high‐dose chemotherapy, followed by autologous hematopoietic stem‐cells transplantation (ASCT), has improved median overall survival (OS) by 6–8 years in newly diagnosed MM (NDMM) patients eligible for ASCT. Currently, 50% of MM patients are still alive 5 years after the diagnosis, and one third of patients live more than 10 years, according to disease risk staging and cytogenetic abnormalities (revised international staging system [R‐ISS]) that have an impact on prognosis [3, 4, 5]. Nevertheless, despite the initial response after first line treatment, most of them experience relapse. Therefore, MM remains an incurable disease, notwithstanding improvements in the MM outcome and in the depth and response duration following subsequent lines of therapy. The reduced sensitivity of neoplastic plasma cells to subsequent drugs causes the emergence of resistant clones, ultimately responsible of patient refractoriness to all available agents. Currently, there is great concern about relapsed/refractory MM (RRMM) due to its pathophysiology mechanisms and clinical complexity.

To overcome the occurrence of relapse, ongoing challenges are warranted to identify novel therapeutic strategies based on different mechanisms of actions. Over the last years, scientific research has been focused on immunotherapy, either passive, as in the case of monoclonal antibodies (MoAbs) or active, that has a pivotal role in the treatment of MM [6, 7]. MoAbs have entered clinical practice for the current treatment of MM, thanks to the highest expression of MoAbs‐therapeutic target on clonal plasma cells surface. Daratumumab is a first in class IgG1k MoAb that targets CD38, which is highly expressed on aberrant plasma cells and other hematopoietic cell surface [8, 9]. Daratumumab shows direct and indirect anti‐tumor activity and the specific mechanisms of action comprise immune‐mediated effects, such as complement‐dependent and antibody‐dependent cell‐mediated cytotoxic effects, antibody‐dependent cellular phagocytosis, and apoptosis by means of cross‐linking [10, 11, 12, 13, 14]. Moreover, daratumumab presents an immumodulatory function that targets and depletes CD38 positive regulator immune suppressor cells, which leads to T cells clonal expansion and activation in MM patient who have a hematological response to treatment [15]. Initial approval by Food and Drug Administration (FDA) and European Medicines Agency (EMA) of daratumumab as monotherapy for MM patients heavily treated (at least 3 lines of therapy) was based on the phase I and II GEN501 and SIRIUS trials [16, 17]. Subsequently, daratumumab in combination with standard of care regimen showed clinical benefit in two different phase III trials involving patients with relapsed or refractory myeloma. The addition of daratumumab to the standard of care regimen, including bortezomib and dexamethasone (CASTOR trial), or lenalidomide and dexamethasone (POLLUX trial), was associated with a significantly prolonged progression free survival (PFS), lower risk of disease progression, or death, a higher percentage of overall response rate (ORR), and minimal residual disease negativity (MRD) with a good safety profile [17, 18]. This led to approval by FDA and EMA for pts with RRMM, as well as in newly diagnosed MM.

We report the experience of the multiple myeloma Lazio group in 171 heavily treated patients treated in real‐life with daratumumab, in combination with bortezomib (DVd) or with lenalidomide plus dexamethasone (DRd).

## 2 METHODS

Between March 2018 and November 2020, 171 consecutive RRMM patients aged >18 years treated with DRd or DVd in 11 hematology departments of multiple myeloma Lazio group were enrolled in a retrospective observational study.

Daratumumab in association with Rd was administered weekly (16 mg/Kg intravenously [iv]) for 8 weeks during cycles 1 and 2; every 2 weeks for 16 weeks (cycles 3 through 6), and every 4 weeks thereafter. All patients received lenalidomide (25 mg orally) on days 1–21 of each cycle and dexamethasone (40 mg) weekly. Conversely, daratumumab in association with velcade and dexamethasone (VD) was administrated once per week during cycles 1–3, once every 3 weeks during cycles 4–8, and once every 4 weeks thereafter. Bortezomib was administered (21 days per cycle) at a dose of 1.3 mg/m^2^ (on days 1, 4, 8, 11) and dexamethasone (40 mg) weekly. The first daratumumab infusion was 1000 ml at 50 ml/h, followed by dose escalation. If the first dose infusion was well tolerated, subsequent infusions were 500 ml at 100 ml/h. Standard premedication was used: methylprednisolone 75–100 mg iv for the first and second infusions, and 60 mg thereafter, or equivalent steroid, paracetamol 1000 mg, antihistamine drug, and/or montelukast, according to the clinical practice.

The dose of each drug was adjusted, according to the drug recommendations, in the presence of specific patient's comorbidities.

Patients received either bortezomib or lenalidomide combined with daratumumab at the discretion of physician.

An efficacy assessment was performed on day 1 of each cycle according to the International Myeloma Working Group (IMWG) criteria [19].

The safety assessment was based on evaluation of hematological and nonhematological toxicity, and all adverse events were recorded using the Common Terminology Criteria for Adverse Events version 5.0.

The primary endpoint of this observational and retrospective study was to evaluate safety and efficacy of daratumumab‐based treatment, in terms of toxicity, ORR, PFS, and OS. Response to therapy was assessed according to the IMWG criteria [13], and an ORR was calculated considering the achievement of at least a partial response (PR).

Patients’ characteristics were compared by chi‐squared or Fisher's exact test for categorical variables. Differences in distributions were assessed by Wilcoxon test for continuous data. Survival curves were estimated by the Kaplan–Meier product‐limit method and compared using the log‐rank test. Cox proportional hazards regression models were used in univariate analyses to estimate the risk on survival outcomes.

All tests were two‐sided, accepting *p* < .05 as index of statistical significance. All analyses relied on the R software. Study data were collected and managed using REDCap electronic data capture tools hosted at the Sapienza University [20, 21].

## 3 RESULTS

The baseline characteristics of the 171 patients are summarized in Table 1. At daratumumab initiation, 91 pts (53%) were male, and 80 pts (47%) were female. Median age was 64 years (range 37–83), median level of hemoglobin was 10.9 g/dl (6.9–16.7), and median level of creatinine was 0.9 mg/dl (range 0.4–7.4). Of 171 pts, 108 (71%) had bone lytic lesions. Cytogenetic features were not available. According to the ISS, 71 pts (48%) were I, 41 pts (28%) were II, and 36 pts (24%) were III. One‐hundred twenty pts (70%) had previously received a single line of therapy; 32 pts (19%) had received two lines of therapy, and 19 pts (11%) had received ≥3 lines of therapy. Median number of previous treatments was 1. Of 171 patients, 78 (90%) had received ASCT as a first line of therapy and nine (10%) as a second line. Eighty‐five pts (49%) had received therapy with IMiDs; 78 pts (45%) therapy with PIs and 8 pts (4.67%) had received therapy with other chemotherapy. Median time from the diagnosis to treatment with daratumumab was 41 months.

**TABLE 1:** Patient's baseline characteristics and previous therapy

	Overall, *N* = 171	DRd, *N* = 133	DVd, *N* = 38
**Median age, years (range)**	64 (37–83)	64 (38–79)	64 (37–79)
**Male, *n* (%)**	91 (53)	69 (52)	22 (58)
**Female, *n* (%)**	80 (47)	64 (48)	16 (42)
**eastern cooperative oncology group (ECOG) 0, *n* (%)**	139 (89)	112 (90)	27 (87)
**ISS III, *n* (%)**	36 (24)	26 (22)	10 (33)
**Bone lesions, *n* (%)**	108 (71)	85 (72)	23 (66)
**hemoglobin (Hb), median (range)**	10.9 (6.9–16.7)	11 (6.9–15.9)	10.5 (8–16.7)
**Creatinine, median (range)**	0.9 (0.4–7.4)	0.9 (0.4–5.3)	0.9 (0.54–7.4)
**Clearance creatinine <60** **ml/min/1.73m^2^**	21	15	6
**lactate dehydrogenase (LDH), median (range)**	191 (72–574)	189 (72–574)	266 (114–429)
**ASCT I° line, *n* (%)**	78 (46)	61 (88)	17 (94)
**ASCT II° line, *n* (%)**	9 (5)	8 (12)	1 (5,6)
**Median number of previous lines of therapy, *n* (range)**	1 (1–5)	1 (1–5)	1 (1–5)
**Median time from diagnosis to starting DRd−DVd therapy (months), *n* (range)**	41 (3–305)	37 (3–221)	52 (3–305)
**Daratumumab line of therapy, *n* (%)**			
**II**	120 (70)	105 (79)	15 (39)
**III**	32 (19)	17 (13)	15 (39)
**IV+**	19 (11)	11 (8.3)	8 (21)
**Proteasome inhibitors exposed, *n* (%)**	146 (85)	125 (93)	21 (55)
**Immunomodulatory drugs exposed, *n* (%)**	100 (58)	65 (49)	35 (92)

Abbreviations: ASCT, autologous hematopoietic stem‐cells transplantation; ISS, international staging system.

One‐hundred thirty‐three (78%) pts received DRd and 38 pts (22%) DVd. Treatment usually continued until progression, unacceptable toxicity, or death, and the median number of cycles was 8 (range 1–30). Of 171 pts, 163 (95%) pts completed at least one cycle of therapy and were evaluated for hematological response. The ORR was 84% (137 pts), specifically, three pts (1.8%) obtained an stringent complete response (sCR), 15 pts (9.2%) a complete response (CR), 34 pts (21%) a very good partial response (VGPR), and 85 pts (52%) a PR (Table 2).

**TABLE 2:** Treatment response on 163 evaluable patients

	Overall, *N* = 163 (%)	DRd, *N* = 126 (%)	DVd, *N* = 37 (%)
**Overall response rate**	137 (84)	110 (87)	27 (72)
**sCR**	3 (2)	3 (2)	0
**CR**	15 (9)	13 (10)	2 (5)
**VGPR**	34 (21)	27 (21)	7 (19)
**PR**	85 (52)	67 (53)	18 (49)
**VGPR or better**	52 (38)	43 (34)	9 (24)
minimal response (**MR**)	10 (6)	7 (5)	3 (8)
stable disease (**SD**)	6 (4)	3 (2)	3 (8)
**Progressive disease**	10 (6)	6 (5)	4 (11)
**Median time to response, months (range)**	3 (1–12)	3 (1–12)	3 (1–12)

Interestingly, 52 pts (38%) obtained at least a VGPR, among these, 43 (83%) had received DRd treatment. Instead, 83/111 pts (75%) obtaining hematological response lower than VGPR were treated with DVd. The median time to achieve at least a PR from starting therapy was 3 months (range: 1–12). According to daratumumab lines of therapy, no significant statistical difference (*p* = .68) was found in ORR of pts that received daratumumab as second lines of therapy, third lines of therapy, and fourth or more lines of therapy.

After a median follow‐up from starting therapy of 13.49 months (range 1–29.73), 10 pts (6%) presented progression disease (PD) during therapy with daratumumab. Globally, 30 pts (18%) presented PD after daratumumab therapy, and, of these, 20 (15%) pts were in treatment with DRd and 10 (26%) with DVd, with no statistical difference (*p* = 0.14).

We further analyzed factors associated with ORR. No significant statistical correlation was found with gender, age, ISS score, median level of hemoglobin, number of previous lines of therapy, and type of therapy, at the time of starting daratumumab therapy. Instead, a statistical correlation was found with ECOG at the time of starting therapy. Specifically, 86% of pts in VGPR presented a good performance status (ECOG 0), unlike 33% of pts that achieved a hematological response lower than VGPR (*p* = 0.031). The number of cycles of therapy with DRd or DVd was found to positively impact on ORR (Table S3).

**FIGURE 1:**
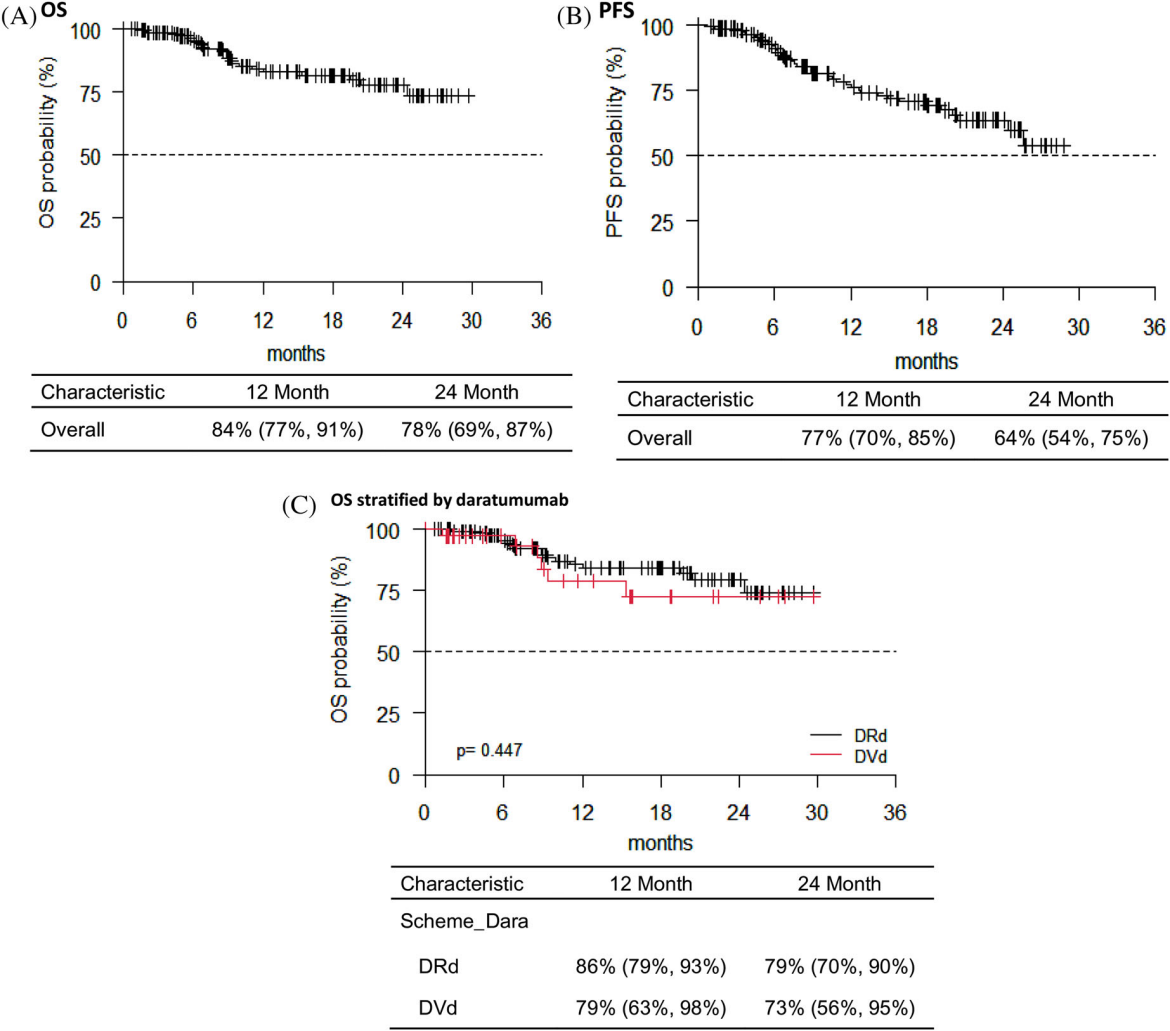
(A) Overall survival (OS) of the entire cohort of patients from daratumumab starting therapy. (B) Progression‐free survival (PFS) of the entire cohort of patients from daratumumab starting therapy. (C) OS of the entire cohort of patients from daratumumab starting therapy stratified according to different scheme of therapy. No statistical difference was found (*p* = .447)

**FIGURE 2:**
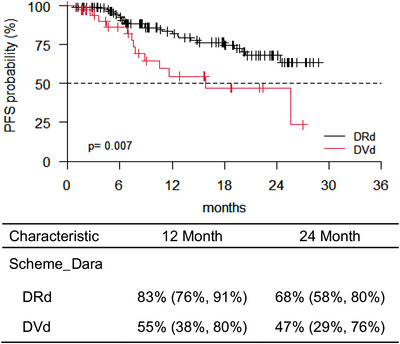
Progression‐free survival (PFS) of the entire cohort of patients from daratumumab starting therapy stratified according to scheme of therapy. Patients (pts) treated with DRd showed a better median PFS compared to pts treated with DVd (*p* = .007)

Globally, OS was 84% (95% confidence interval (CI), 77–91) at 12 months and 78% (95% CI, 69–87) at 24 months, respectively. In addition, PFS was 77% (95% CI, 70–85) at 12 months and 64% (95% CI, 54–75) at 24 months, respectively. We analyzed median OS stratified, according to type of treatment, and no statistical difference was found at 12 or 24 months (*p* = 0.447) (Figure 1). Conversely, pts treated with DRd showed a better median PFS compared to pts treated with DVd, at 12 and 24 months, respectively (*p* = 0.007) (Figure 2). No statistical difference was found in PFS at 12 and 24 months respectively, of pts treated with DRd or DVd stratified, according to the number of previous lines of therapy (*p* = .259 and *p* = .652 for II and III line of therapy, respectively) (Figures S3 and S4). According to the number of lines of therapy, pts treated with daratumumab at II line had a better PFS at 12 and 24 months, respectively, compared to pts treated with ≥3 lines of therapy with daratumumab (*p* = .003) (Figure S5). Finally, no statistical difference was found in OS at 12 and 24 months, respectively, according to the lines of therapy (*p* = .152) (Figure S6).

The elevated LDH at diagnosis, a bad performance status either at diagnosis or at the time of starting therapy with daratumumab, and anemia were found to negatively affect the OS. In addition, the reduced level of white blood cell and elevated level of LDH at diagnosis, anemia, and a bad performance status at the time of starting therapy with daratumumab were found to negatively affect the PFS. We further demonstrated that a DVd scheme of therapy, a hematological response lower than VGPR, and daratumumab in fourth, or more lines of therapy, negatively influence the PFS.

All patients included were analyzed on safety. The most common hematologic treatment‐emergent adverse events (TAEs) were neutropenia (60%), thrombocytopenia (32%), and anemia (18%). Hematologic grade 3–4 toxicities included neutropenia (52%), thrombocytopenia (30%), and anemia (14%). Of 50 pts with hematologic TAEs, 24 pts (48%) had reduced the dosage of lenalidomide (42%) or bortezomib (6%). Six patients (12%) had a delayed lenalidomide or bortezomib dose, and 10 pts (20%) had discontinued lenalidomide therapy. Of these 50 pts, six pts (12%) had permanently discontinued DRd (four pts) or DVd (two pts) treatment.

Nonhematologic TAEs were identified in 77 pts (47%), and of these 77 pts, 70 had nonhematologic events of grade 2–3. Only two pts had adverse events of grade 4. Specifically, infections occurred in 22 pts (28%), eight pts had pneumonia, and three pts had viral reactivations. All pts received antibacterial and antiviral prophylaxis therapy, according to the clinical practice and the physician's choice. All the patients also received *Pneumocystis carinii* prophylaxis therapy. Besides infection complications, the second common group of nonhematologic TAEs was peripheral neuropathy that was observed in 15 pts (19%). Atrial fibrillation occurred in one pt, and seven pts (9%) presented diarrhea. The rate of deep venous thrombosis was 3.89%. We observed two cases of secondary primary malignancies, specifically breast cancer.

Out of 77 pts with nonhematologic TAEs, 18 pts (23%) had reduced lenalidomde (17 pts) or bortezomib (one pt) dosage. Six pts (8%) had delayed lenalidomide or bortezomib dose, and eight pts (10%) had permanently interrupted lenalidomide therapy. Moreover, nine pts (11%) had permanently discontinued DRd, or DVd treatment.

The most common infusion‐related reactions (IRRs) were dyspnea, nasal congestion, cough, and rash. No grade 3–4 IRRs were observed, and no pts had discontinued DRd, or DVd therapy, due to IRRs complications.

## 4 DISCUSSION

This retrospective analysis evaluated real‐life data, particularly efficacy and safety, of 163 RRMM pts treated with DRd or DVd in 11 hematology departments of the multiple myeloma Lazio group. In two phase III trials, daratumumab was evaluated in combination with various standards of care, PI and IMiDs, respectively. Specifically, the phase III POLLUX trial, with primary endpoint PFS, compared DRd versus Rd in 569 pts with RRMM who had previously received ≥1 line of therapy [18]. Updated data, after a median follow‐up of 54.8 months, showed a significantly longer PFS for pts treated with DRd compared to those treated with Rd with a 56% reduction in the risk of progression or death (median 45 months vs. 17.5 months, *p* < .0001). In addition, in pts with one prior line of therapy, the PFS was longer in DRd arm versus Rd arm (53.3 months vs. 19.6 months, *p* < .0001) [22]. According to the rate of response, a significantly higher ORR was observed with DRd versus Rd (93% vs. 76%), including ≥VGPR (81% vs. 49%). The most common adverse events occurred in pts treated with DRd were neutropenia, thrombocytopenia, and infections. In the phase III CASTOR trial, daratumumab was studied in combination with VD, and compared to pts treated with only Vd [17]. This trial included 498 RRMM pts who had previously received a median of two lines of therapy. After a median follow‐up of 50.2 months, updated results showed a PFS significantly longer in DVd group versus Vd (16.7 months vs. 7.1 months, *p* < .0001), and this benefit appeared especially in pts receiving DVd as first relapse therapy (median PFS 27 months vs. 7.9 months, *p* < .0001). The ORR was higher in the DVd group compared to the Vd group (85% vs. 63%), as the number of pts achieved VGPR or better (63% vs. 29%, *p* < .0001). The most common adverse events were neutropenia, thrombocytopenia, infections, and neuropathy. As observed in the POLLUX trail, the IRRs occurred during the first infusion of daratumumab, and no grade 3 or grade 4 was detected [23]. The significant results, in terms of ORR and PFS, reported in these two studies supported the use of daratumumab in combination with bortezomib, or lenalidomide, to obtain a deep response and prolonged PFS in RRMM pts. More recently, daratumumab was approved by FDA and EMA, in combination with lenalidomide and dexamethasone (MAYA trial) [24], or in combination with bortezomib, velcade, melphalan and prednisone (VMP) (ALCYONE trail) [25] for newly diagnosed MM pts who were considered ineligible for ASCT. Among these pts, the risk of death or disease progression was significantly lower when daratumumab was added to Rd or VMP.

Compared to POLLUX and CASTOR clinical randomized trials enrolled pts, our retrospective series of pts showed similar clinical‐biological characteristics. Our results were consistent with previous literature data with a high ORR (80%) showed in heavily pts treated with daratumumab in combination with lenalidomide or bortezomib [22, 23]. The lower number of our series of pts that obtained a VGPR or better, compared with the pts enrolled in the other clinical trials, could be explained by the less selected series population of our study with more heavily pretreated pts compared to clinical trial. In addition, we showed that the high ORR was obtained regardless of ISS, gender, age, median level of hemoglobin, and number of previous lines of therapy. In our real‐life retrospective study, we observed a lower rate of CR and sCR, compared to the clinical trials, without worsening the PFS. Given the widely availability of new therapeutic options to treat RRMM, there is an important concern about real‐life experience treatment. Several data showed daratumumab monotherapy efficacy in heavily treated pts in real‐life experience [26, 27, 28]. Data regarding daratumumab combination therapy outside of clinical trials remain limited. A report from Mayo clinic showed that OS and PFS rates in heavily pretreated patients were lower than those reported in clinical trials [29]. Recently two studies [30, 31] have showed data about efficacy and safety of DRd and DVd in real‐life setting. However, in these two studies, the ORR was evaluated considering all the daratumumab‐treated population, without focusing specifically on daratumumab in combination with PI or IMiDs. Antonioli et al. reported a real‐life single‐center experience on RRMM treated with DRd [32]. The authors reported a rate of ORR of 79% with CR observed in 13 pts with a 1‐year PFS and OS rate of 70% and 81%, respectively. These data were consistent with our analysis, except for the higher rate of CR compared to our study. Nevertheless, a higher number of our pts were exposed to lenalidomide as induction or maintenance therapy compared to Antonioli's study. The analysis of the safety data showed that DRd or DVd was well tolerated with a low rate of treatment discontinuation due to toxicity (15 pts). The rate and type of TAEs and IRRs were positively consistent with the known safety profile of DRd or DVd treatment, and no new adverse events were reported.

Considering the available therapy armamentarium and options for RRMM to date, our data support the treatment with DRd or DVd for heavily pretreated pts. This real‐life analysis showed that daratumumab in combination with lenalidomide or bortezomib, plus dexamethasone is a highly effective and well‐tolerated regimen to be considered for RRMM pts after first relapse, producing a high level of ORR and a prolonged PFS and OS. It is important to point out that this real‐life experience was not directly comparable with previously published randomized clinical trial results, but we believe that our data have a positive clinical impact on real‐life treatment choice in RRMM pts and support physicians in the choice of standard clinical therapy.

## AUTHOR CONTRIBUTIONS

Francesca Fazio drafted the manuscript. Francesca Fazio, Luca Franceschini, Valeria Tomarchio, Angela Rago, Maria Grazia Garzia, Luca Cupelli, Velia Bongarzoni, Alessandro Andriani, Svitlana Gumenyuk, Agostino Tafuri, Agostina Siniscalchi, Paolo De Fabritiis, Luca De Rosa, Tommaso Caravita, Ombretta Annibali, Maria Cantonetti, and Maria Teresa Petrucci made therapeutic decision about the patient and followed the clinical course of patient during chemotherapy. Alfonso Piciocchi analyzed the data; all authors reviewed and approved the final versions of the manuscript.

## CONFLICT OF INTEREST

The authors declare that there is no conflict of interest that could be perceived as prejudicing the impartiality of the research reported.

## Supporting information

Supporting information
