# Supplementary material for: Daratumumab combined with dexamethasone and lenalidomide or bortezomib in relapsed/refractory multiple myeloma (RRMM) patients: Report from the multiple myeloma GIMEMA Lazio group
Source: EJHaem. 2022 Jan 15;3(1):121–8. doi: 10.1002/jha2.359 (PMC9175681; doi:10.1002/jha2.359)
Supplement: Supplementary file 1 — Supporting information [file JHA2-3-121-s001.docx]

**Supplemental Table 3. Factors associated with ORR**

| Characteristics | HR^1^ | 95% CI^1^ | p-value | q-value^2^ |
| --- | --- | --- | --- | --- |
| **Scheme Daratumumab** |  |  | 0.46 | 0.89 |
| DRd | — | — |  |  |
| DVd | 1.43 | 0.56, 3.64 |  |  |
| **Sex** |  |  | **0.041** | 0.23 |
| Male | — | — |  |  |
| Female | 2.38 | 1.01, 5.63 |  |  |
| **Age at diagnosis** | 1.00 | 0.96, 1.04 | 0.85 | 0.89 |
| **Daratumumab line of therapy** |  |  | 0.21 | 0.59 |
| II | — | — |  |  |
| III | 1.60 | 0.61, 4.20 |  |  |
| IV + | 2.83 | 0.92, 8.69 |  |  |
| **Number of cycles of therapy** | 1.01 | 0.94, 1.08 | 0.82 | 0.89 |
| **Age at starting daratumumab** | 0.99 | 0.95, 1.04 | 0.78 | 0.89 |
| **ECOG at starting daratumumab** | 3.11 | 1.03, 9.38 | **0.073** | 0.27 |
| **ISS at starting daratumumab** |  |  |  |  |
| I | — | — |  |  |
| II | 0.59 | 0.19, 1.86 |  |  |
| III | 1.15 | 0.40, 3.31 |  |  |
|  |  |  |  |  |
| **Creatinine at starting daratumumab** | 0.94 | 0.53, 1.65 | 0.81 | 0.89 |
| **Hb at starting daratumumab** | 0.68 | 0.54, 0.86 | **<0.001** | 0.009 |
|  |  |  |  |  |
|  |  |  |  |  |
|  |  |  |  |  |
|  |  |  |  |  |
|  |  |  |  |  |

### Supplemental Figure 3

### PFS stratified by Daratumumab in line II


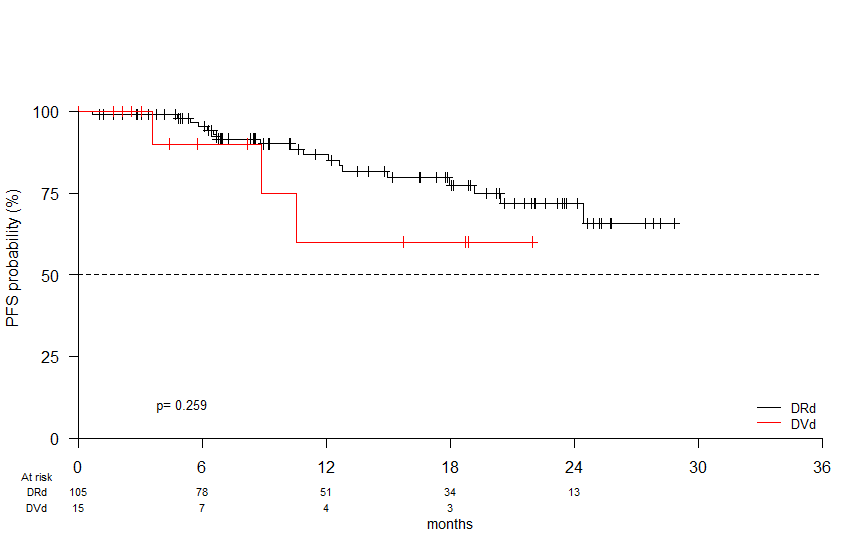


| Characteristic | 12 Month | 24 Month |
| --- | --- | --- |
| Scheme_Dara |  |  |
| DRd | 87% (79%, 95%) | 72% (60%, 85%) |
| DVd | 60% (33%, 100%) | — (—, —) |

**3. A** PFS of the entire cohort of patients from daratumumab starting therapy stratified according to scheme of therapy and daratumumab as second line of therapy. No statistical difference was found (p=0.259).

### Supplemental Figure 4

### PFS stratified by Daratumumab in line III


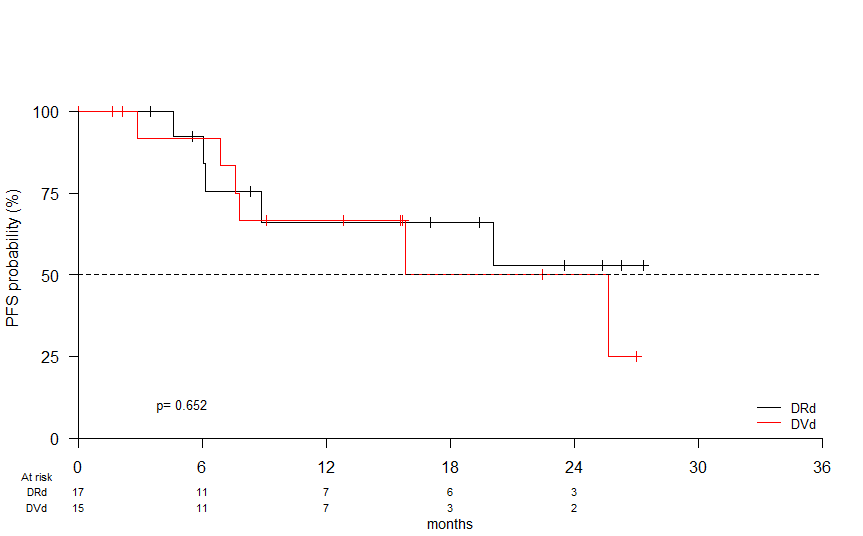


| Characteristic | 12 Month | 24 Month |
| --- | --- | --- |
| Scheme_Dara |  |  |
| DRd | 66% (44%, 100%) | 53% (29%, 97%) |
| DVd | 67% (45%, 99%) | 50% (25%, 100%) |

###

**4. A** PFS of the entire cohort of patients from daratumumab starting therapy stratified according to scheme of therapy and daratumumab as third line of therapy. No statistical difference was found (p=0.652).

### Supplemental Figure 5.

### PFS stratified by Daratumumab line


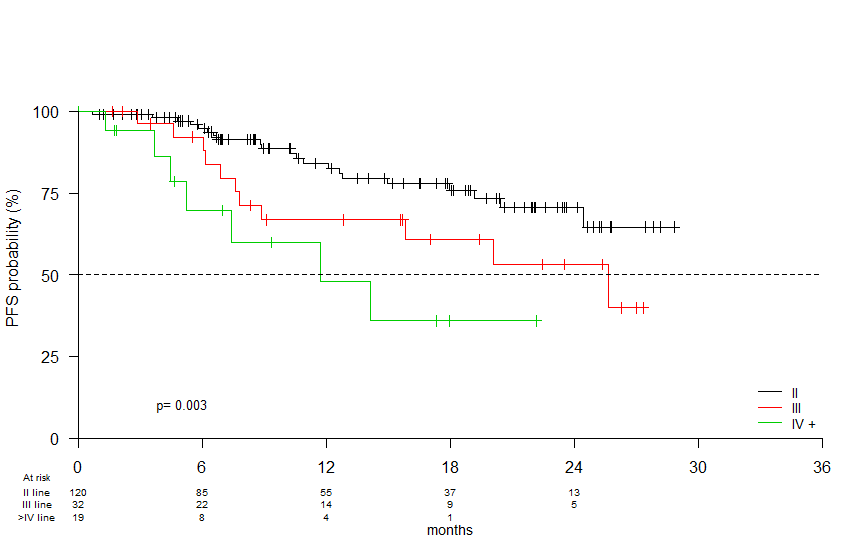


| Characteristic | 12 Month | 24 Month |
| --- | --- | --- |
| dara |  |  |
| II | 84% (76%, 93%) | 70% (60%, 83%) |
| III | 67% (50%, 89%) | 53% (35%, 82%) |
| IV + | 48% (25%, 91%) | — (—, —) |

**5 .A** PFS of the entire cohort of patients from daratumumab starting therapy according to the number of lines of therapy. Pts treated with daratumumab at II line had a better PFS compared to pts treated with ≥ 3 lines of therapy with daratumumab (p=0.003).

###

### Supplemental Figure 6.

### OS stratified by Daratumumab line


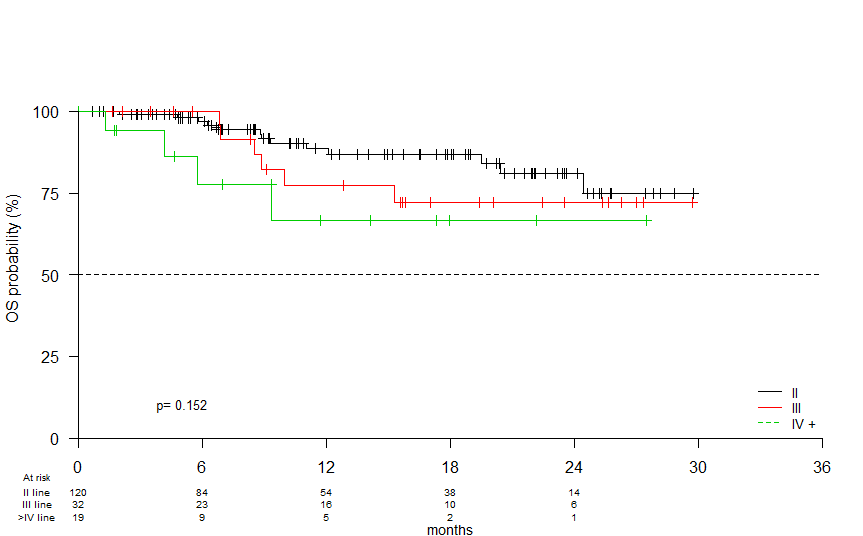


| Characteristic | 12 Month | 24 Month |
| --- | --- | --- |
| dara |  |  |
| II | 89% (82%, 96%) | 81% (71%, 92%) |
| III | 77% (62%, 97%) | 72% (55%, 94%) |
| IV + | 67% (44%, 100%) | 67% (44%, 100%) |

**6**. OS of the entire cohort pf patients from daratumumab starting therapy according to the lines of therapy. No statistical difference was found (p=0.152).
